# High dynamic resistance elements based on a Josephson junction array

**DOI:** 10.3762/bjnano.11.32

**Published:** 2020-03-03

**Authors:** Konstantin Yu Arutyunov, Janne Samuel Lehtinen

**Affiliations:** 1 National Research University Higher School of Economics, 101000, Moscow, Russia; 2 P.L. Kapitza Institute for Physical Problems RAS, Moscow, 119334, Russia; 3 VTT Technical Research Centre of Finland Ltd., 02150 Espoo, Finland; 4 Department of Physics, University of Jyvaskyla, PB 35, FI-40014 Jyvaskyla, Finland

**Keywords:** dynamic resistance, Josephson junction array, nanoelectronics, quantum phase slip, superconductivity, Ti nanowires

## Abstract

A chain of superconductor–insulator–superconductor junctions based on Al–AlO*_x_*–Al nanostructures and fabricated using conventional lift-off lithography techniques was measured at ultra-low temperatures. At zero magnetic field, the low current bias dynamic resistance can reach values of ≈10^11^ Ω. It was demonstrated that the system can provide a decent quality current biasing circuit, enabling the observation of Coulomb blockade and Bloch oscillations in ultra-narrow Ti nanowires associated with the quantum phase-slip effect.

## Introduction

The field of modern nanoelectronics is facing stagnation with respect to further miniaturization, deviating from Moore’s law [1]. Typically, two main reason are quoted: severe heat dissipation per unit volume (surface), and various quantum phenomena that drive the operation of ultra-small devices and make them different from devices in the conventional (classical) regime. The radical solution to the first problem is to build the critical elements using superconductors. The basics of this approach were developed in the late 1980s, resulting in rapid single flux quantum (RSFQ) logic [2]. Since that time the concept has continued to develop. However, the corresponding systems so far have not developed into mass market commercial products, being limited solely to particular “cost-no-object” applications. Currently, the field of superconducting electronics is developing much faster mainly due to the understanding that (even taking into consideration the necessity of refrigeration) the energy consumption of next generation supercomputers can be as low as ≈10 MW, which is compared to values of ≈100 MW for conventional semiconductor complementary metal–oxide-semiconductor (CMOS) technology. In addition to heat dissipation, another issue is the speed of processing. It has been shown that the operational frequency of superconducting logic can be at least 100 times higher than for CMOS-based devices. It is universally accepted that the limiting factor for the speed of operation of various superconducting devices is the high-frequency impedance, e.g., originating from kinetic inductance. The effect should be taken into consideration for various cryoelectronic applications.

In addition to RSFQ computers which exploit classical 2-bit logic, during the last decades, there has been an increasing interest in quantum computing utilizing nonclassical approaches. There have been multiple suggestions regarding how to build quantum logic elements, such as quantum bits (qbits), including superconducting systems based on the Josephson effect. It has been shown that physics behind a Josephson junction (JJ) is dual to a quantum phase-slip junction (QPSJ) [3], whereby the corresponding QPSJ-based qbit operation has also been demonstrated [4]. At the same time, the quantum dynamics of a JJ (or a QPSJ) is strongly determined by the environment [5–6]. In particular, the utilization of devices based on quantum fluctuations of the macroscopic phase, φ, requires stabilization of the quantum conjugated quantity – charge *q*. The most straightforward approach is to use high-Ohmic on-chip current-biasing elements [7–10]. However, it was later understood that resistive dissipative elements inevitably act as a source of Johnson noise, leading to degradation of system performance [11].

Here we present an experimental study of a quasi-1D chain of JJs. A sufficient high-frequency impedance was demonstrated to study the QPS phenomena without the undesired impact of Johnson noise typically associated with dissipative elements [12]. The *I*–*V* dependence studied in [12] demonstrated clear and expected characteristics at low current, *I* → 0: the so-called “Bloch nose” (back-bending of *I*–*V*), while at finite current values, the corresponding singularities were not so pronounced. The purpose of this paper is to provide an in-depth analysis of the *I*–*V* dependence of the same JJ chains used in the current-biasing elements in [12].

## Experimental

**Figure 1:**
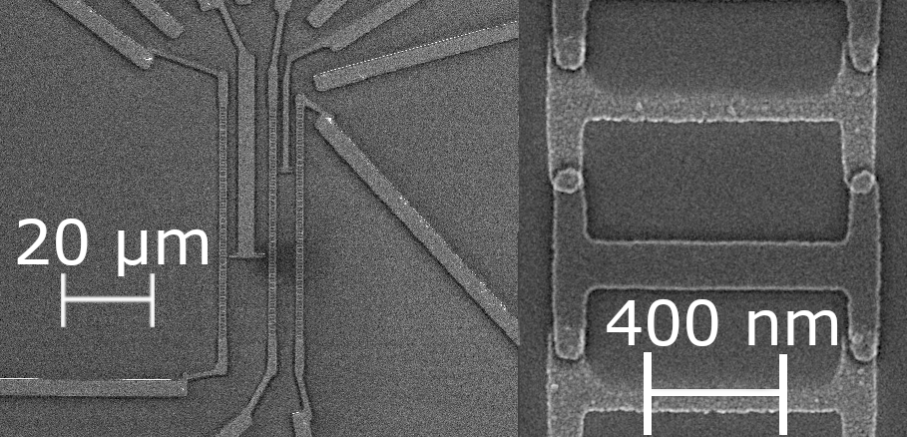
SEM images of the test sample fabricated from superconducting thin film aluminum oxidized in situ to form tunnel barriers. Left panel: overview of the structure. Right panel: details of the JJ element.

Conventional lift-off electron-beam lithography followed by ultrahigh vacuum deposition of materials was used for the fabrication of the nanostructures. Hybrid QPSJ samples were made of Ti, Al and aluminum oxide [12]. The high-impedance JJs studied in this paper, similar to those from [12], were fabricated from superconducting thin film Al oxidized in situ to form tunnel barriers. Each sample consisted of 25 pairs of JJs connected in parallel where the area of each superconductor–insulator–superconductor (SIS) contact was about 100 × 100 nm (Figure 1). The samples were analyzed by scanning electron microscopy (SEM) (Figure 1) and atomic force microscopy (AFM).

Transport measurements were made inside a ^3^He^4^He dilution refrigerator at temperatures below 400 mK, corresponding to the superconducting transition of Ti QPSJs [10,12]. All input/output lines were carefully filtered [13] to reduce the impact of the noisy electromagnetic environment. When necessary, a small magnetic field, up to 0.05 T, was applied using small superconducting coils wound directly on the sample holder cap.

## Results and Discussion

The ultimate goal of this work is to study the quantum dynamics of the QPSJ, a system dual to JJ [3], including the observation of Coulomb blockade and Bloch oscillations [14]. Given that the macroscopic phase, φ, and the charge, *q*, are quantum conjugated values, [φ̂,*q̂*] = *ih*, in order to enable the high rate of phase fluctuations, one should define the charge. The phase–charge duality in conventional JJ systems is well established [15–17]. Hence, to enable the phase fluctuation regime, the electric current, *I*, through a QPSJ, which is just the time derivative of charge, *I =* d*q*/d*t*, should be stabilized. The focus of this manuscript is to study the transport properties of JJ chains to be used as current-biasing elements of a QPSJ. Note that here the finite electric current is maintained by correlated Cooper pair tunneling at a voltage bias *V* across the QPSJ exceeding the particular Coulomb blockade threshold, *V*_C_ [14]. The tunneling happens at the Bloch oscillation rate, *f*_B_. The synchronization of this “internal” periodic process with the external drive, *f*_RF_, should result in quantized singularities (Bloch steps) at current values *I*(*n*) = *n*(2*e*)*f*_RF_, where 2*e* is the charge of the Cooper pair and *n* = 1,2,3... are integers. Furthermore, the study of QPSJ *I–V* characteristics demonstrating Coulomb blockade at zero current, *I* = 0, and voltages of *V* < *V*_C_ requires only the high-Ohmic environment with resistance *R*_env_ exceeding the quantum value *R*_env_ > *R*_Q_ = *h*/*e*^2^ ≈ 26 kΩ. While at finite current values, *I* > 0, one needs current stabilization at high frequency *f*_RF_, which further requires high values of the high-frequency impedance, *Z*_env_(*f*_RF_). The observation of a pronounced Coulomb blockade has been observed in JJs using both a high-resistive dissipative environment [7–8] and nonlinear Josephson elements with high dynamic resistance and/or kinetic inductance [6,18]. However, extended attempts to observe Bloch oscillation phenomena at finite currents in JJs provided rather modest results [7–8,19]. The recent progress in understanding the QPS phenomena [20] in ultra-narrow superconducting channels has revived interest in this topic, resulting in the observation of a decent Coulomb blockade [9–10], while quite blurred Bloch steps at finite current values have been detected so far [10]. Later it was understood that the straightforward approach of using a high-Ohmic dissipative environment, *R*_env_ > *R*_Q_, is far from optimal, as it introduces Johnson noise, washing out the desired current singularities [11]. Various JJ-based systems were suggested which take advantage of the high kinetic inductance of superconducting quantum interference devices (SQUIDs) [21–22] (*L*_k_ = cos^−1^(Φ/Φ_0_) at a degeneracy point when Φ/Φ_0_ → π/2, where Φ is the magnetic flux through the SQUID area and Φ_0_ is the magnetic flux quantum, Φ_0_ = *h*/2*e* = 2 × 10^−15^ Wb). Hence the SQUID-based approach requires application of a finite magnetic field. Given that the electromagnetic horizon of our QPSJ is of the order of ≈100 μm [23–25], the corresponding high-impedance current biasing circuit should be of appropriate (small) dimensions. Thus the area of the SQUID is small, and hence a magnetic field corresponding to Φ/Φ_0_ → π/2 can easily reach the ≈10 mT range. At such a magnetic field, two undesirable effects might happen both with the biasing superconducting leads and with the QPSJ. Namely, the formation of Abrikosov vortices and a noticeable suppression of the energy gap. Consequently, in our approach, we opted for a non-dissipative (superconducting) high-impedance environment under zero magnetic field.

**Figure 2:**
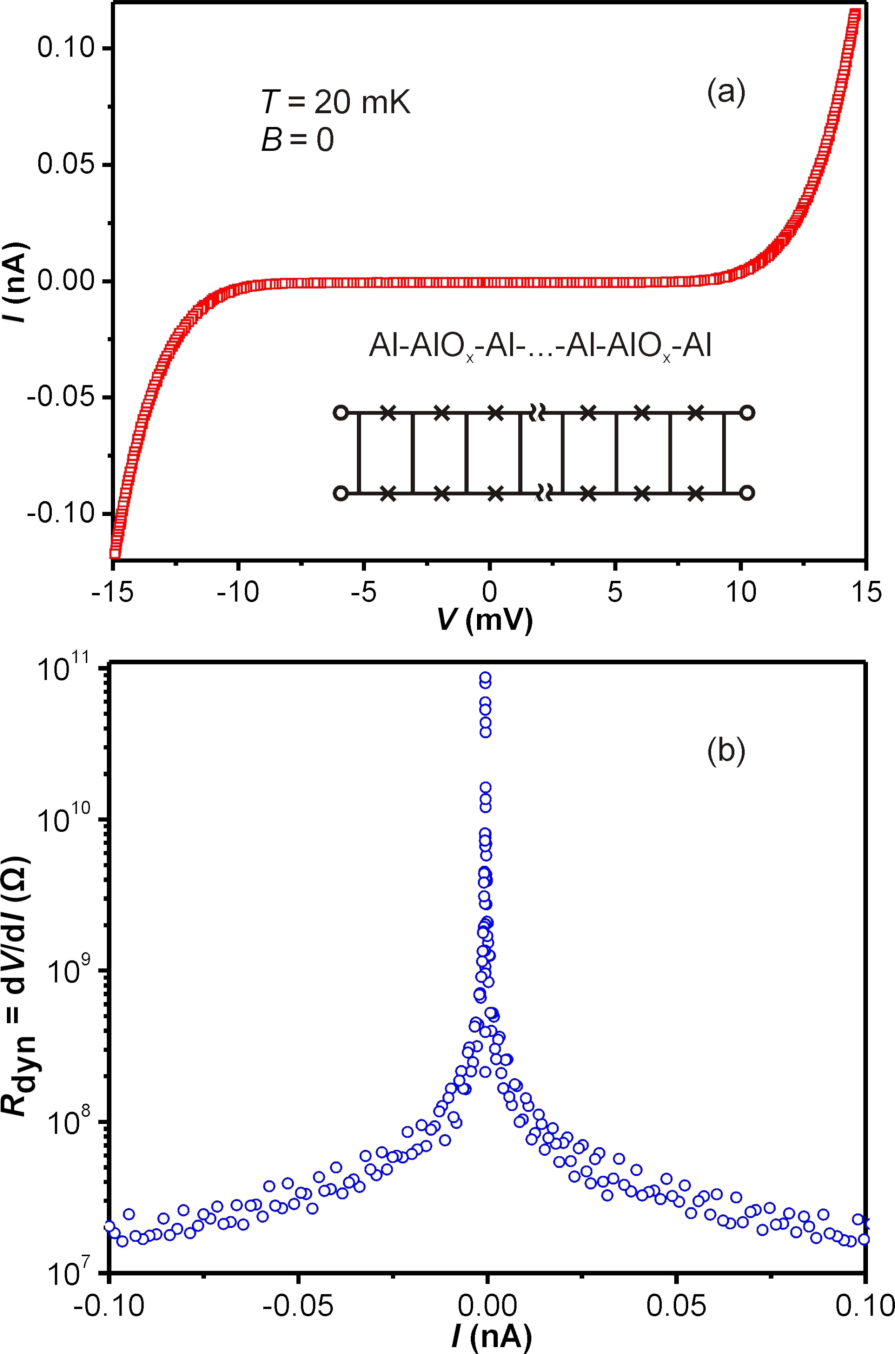
(a) Experimental *I*–*V* characteristics of 50 pairs of Al–AlO*_x_* junctions connected in series. Inset shows a schematic of the structure. (b) Dynamic resistance *R*_dyn_ ≡ d*V*/d*I* obtained by numeric differentiation of the *I*–*V* dependence.

Our quasi-one-dimensional arrays of SIS junctions contain loops forming SQUIDs (Figure 1). The Josephson current is very small (Figure 2a), *I*_c_ < 10 pA, and application of the magnetic field only monotonically suppresses the superconducting gap. The corresponding *I*–*V* dependence can be understood as a tunnel characteristic of multiple SIS junctions connected in series. The *I*–*V* characteristics (Figure 2a) with a gap of ≈10 mV corresponds well with 25 SIS junctions connected in series, each being a Al–AlO*_x_*–Al junction with a gap of about 400 µV. The charging energy, *E*_c_ = e^2^/2C, of each SIS contact (considering it to be a plate capacitor with dielectric constant ε ≈ 10, area 100 × 100 nm and distance between plates ≈2 nm) is about two orders of magnitude higher than the Josephson energy, *E*_J_ = *I*_c_/2e. As *E*_J_ << *E*_c_ the physics of the system is dominated solely by charging phenomena. At zero magnetic field and small current bias, the dynamic resistance *R*_dyn_ ≡ d*V*/d*I* of the JJ chain can reach ≈10^11^ Ω (Figure 2b), while at a higher bias, *R*_dyn_ (*I* >> 0) approaches 100 kΩ.

The SIS junction chain has been used to current bias narrow Ti nanowires [12], with cross sections demonstrating various phenomena attributed to the QPS effect [10,26–33]. The observation of Coulomb blockade and Bloch steps [12] confirms the usefulness of the suggested concept, that is, the utilization of SIS junction chains.

Summarizing, we can conclude that chains of series-connected tunnel SIS junctions can provide high dynamic resistance at low current, which is necessary to stabilize the charge of a quantum circuit and hence enable the high level of phase fluctuations. The absence of dissipation makes such elements very useful for experimental studies of mesoscopic scale objects at ultralow temperature applications, where even a very small amount of Johnson noise may overheat electrons above the phonon bath. However, the non-linearity of a SIS junction *I*–*V* characteristic makes them less useful at finite current biases, which dramatically reduces the dynamic resistance. A promising solution might be the utilization of superconducting circuits with a high level of kinetic inductance capable to provide a sufficient impedance at high frequencies.
